# A Case of Congenital Glaucoma in a 5-Year-Old Patient With Sturge–Weber Syndrome and Oculodermal Melanocytosis

**DOI:** 10.1155/crop/3902349

**Published:** 2025-08-01

**Authors:** Param Shukla, Miriam Habiel

**Affiliations:** Department of Ophthalmology, Rutgers New Jersey Medical School, Newark, New Jersey, USA

**Keywords:** Ahmed tube, glaucoma, oculodermal melanocytosis, pediatric, Sturge–Weber syndrome

## Abstract

**Purpose:** This study was aimed at presenting a case of Sturge–Weber syndrome and oculodermal melanocytosis in a pediatric patient and offering a viable treatment course to control the glaucoma in both eyes.

**Observations:** A 5-year-old female presents with a large port-wine stain on the left side of her face, retinal pigment changes, and diffuse slate gray pigmentation of the sclera, consistent with Sturge–Weber syndrome and oculodermal melanocytosis. She underwent bilateral trabeculotomy, micropulse cyclophotocoagulation, and staged Ahmed tube insertion for the management of her glaucoma. The pressures have normalized bilaterally after tube insertion, with the last measurement of her eyes under anesthesia revealing intraocular pressures of 25 in the left and 18 in the right.

**Conclusions:** It is possible to achieve intraocular pressure control in a patient with congenital glaucoma associated with Sturge–Weber syndrome and oculodermal melanocytosis using staged Ahmed tube insertion.

## 1. Introduction

Sturge–Weber syndrome (SWS) is a rare neurocutaneous disorder that presents with abnormal venous malformations of the skin, eye, and/or brain. The pathophysiology of this syndrome involves a sporadic activating mutation in the GNAQ gene that occurs in one in every 20,000–50,000 live births [1, 2]. The abnormal vasculature typically presents with a facial port-wine stain in the distribution of the trigeminal nerve, which is often unilateral, present at birth, and resistant to change with age [3]. It can also present with choroidal hemangiomas and progressive neurologic problems from leptomeningeal capillary venous malformations [4]. The neurologic problems usually manifest in the form of atonic, tonic, or myoclonic seizures sometimes accompanied by hemiparesis in the territory of the associated venous malformation [3]. Miller noted a 30% incidence of glaucoma in patients with SWS, while Sullivan et al. observed an incidence as high as 71% [5, 6]. Budenz et al. reported that glaucoma is usually seen at birth in about 60% of patients with SWS, while the other 40% of patients end up developing glaucoma later in childhood or adulthood [7]. Specifically, if there is port-wine stain involvement of both the upper and lower eyelids, then the risk of glaucoma in the patient increases by 50% on the ipsilateral side compared to patients with the port-wine stain on only the upper or lower eyelid [3]. Potential ocular symptoms that can develop early in infancy include increased vascularity of the conjunctiva, increased tearing, increased axial length, buphthalmos, myopia, photophobia, and strabismus [3]. This syndrome complicates the management of glaucoma in these patients due to the presence of diffuse choroidal hemangiomas seen in about 20% of the patients on the same side as the facial port-wine stain [8]. This makes potential surgical interventions have an increased risk of a choroidal hemorrhage either during or after the surgery. Children with this disorder require close follow-ups for the screening for glaucoma, amblyopia, and refractive errors, as well as a multidisciplinary approach to address neurological or developmental abnormalities [9].

Oculodermal melanocytosis (ODM) is an asymptomatic congenital pigmentation involving the trigeminal nerve distributions of the face. Most commonly, the V1 ophthalmic and V2 maxillary branches are affected [10]. The characteristic presentation is a gray-blue hyperpigmentation of the sclera and conjunctiva [10]. The two main ophthalmic complications that may arise from ODM are an increased predisposition to uveal melanoma and glaucoma [11]. This condition is common in the Asian population with an incidence of one in 300 [10]. Teekhasaenee et al. previously reported around a 10% prevalence of glaucoma in patients with ODM, with approximately 1.5% who suffered from the congenital variant [12]. Magarasevic and Abazi and Abdolrahimzadeh et al. postulated that open-angle glaucoma develops as a result of this accumulation of pigment in the trabecular meshwork and Schlemm's canal leading to an aqueous humor outflow obstruction [13, 14]. The management of glaucoma in these patients is similar to the treatment in most patients with open-angle glaucoma [11].

The co-occurrence of ODM and SWS is rare, with less than 50 total reported cases in the past 25 years. Here, we report a case of a 5-year-old girl with SWS, ODM, congenital glaucoma, and seizures who has been able to achieve optimal intraocular pressure (IOP) control with surgical interventions.

## 2. Case Report

A 5-year-old girl presented to the pediatric eye clinic at University Hospital in Newark, New Jersey (NJ), to restart treatment for congenital glaucoma in both eyes. She was originally being treated at Emory Eye Center in Atlanta, Georgia, but moved to NJ and did not follow up with eye treatments for the past 2 years. She has a history of developmental delay, epilepsy, and hydrocephalus requiring a ventriculoperitoneal shunt. At birth, she had congenital glaucoma with a blue hue of the corneas noted bilaterally and needed a trabeculotomy in both eyes. Topical treatment with brinzolamide, latanoprost, and timolol was given to her postoperatively for continued IOP management before she relocated to NJ.

General examination revealed a port-wine stain on the left side of the face, including the left upper and lower eyelid. She was started on latanoprost QHS OU, but more definitive decisions regarding her treatment were deferred to the next visit when an eye exam under anesthesia (EUA) could be performed.

During the first EUA, IOP was determined to be 37 in the right eye and 36 in the left. Once again, hyperpigmentation was observed on the left eyelids and surrounding periorbital areas, which is consistent with presentations in SWS. No Frank Haab's striae were observed bilaterally, but there was mild corneal haze. The sclera was observed to have slate gray pigmentation in almost 360° in both eyes, which is consistent with ODM. She was phakic in both eyes.

In the right eye, a supertemporal corneal wound was identified, consistent with prior glaucoma surgery. Angle structures examined using gonioscopy displayed an immature angle with multiple areas of peripheral anterior synechiae. The vitreous was clear, and the nerve was pink and sharp, measuring 0.45 V with an early temporal notch. The retinal vessels appeared tortuous. Additionally, the visible retina was flat with pigmentation changes almost 360, most prominently on the superonasal side.

In the left eye, angle structures examined using gonioscopy displayed an immature angle that was closed temporally, but the remaining 270° appeared open with multiple areas of peripheral anterior synechiae. The vitreous was clear, and the nerve appeared to have slight pallor. It measured 0.75 V with shunt vessels and temporal rim loss. The retinal vessels appeared tortuous. The visible retina was flat with pigmentation changes almost 360 similar to the right eye, but the most prominent pigmentation was inferonasal. The pigmentary changes noted in both eyes in the retina were attributed to myopic fundus changes.

Based on these exam findings and increased IOP, a discussion was had with the mother regarding surgical versus laser options for treatment. She decided to proceed with laser treatment first. A micropulse cyclophotocoagulation (CPC) was performed in both eyes, treating a total of three quadrants (sparing superonasally and 9 and 3 o'clock) for five cycles with 100 s each (20-s sweep), using a power of 2500 mW and a 31.3% duty cycle. Postoperatively, the patient was started on a regimen of latanoprost every night and dorzolamide/timolol twice a day in both eyes along with a short steroid taper.

The patient was then evaluated 7 weeks later with another EUA. IOP at that time was 28 in the right eye and 29 in the left eye. The rest of the exam was similar to previous findings. As a result of the persistently elevated pressures, a decision was made to proceed with Ahmed glaucoma valve insertion. The left eye was operated on first because of the more advanced cupping. Due to the increased risk of choroidal hemorrhage and bleeding during surgery in patients with SWS, the procedure was performed in stages. In the first stage, the plate and tube were placed on the eye subconjunctivally, but the tube was not inserted into the anterior chamber to allow for adequate encapsulation to minimize risks of hypotony postoperatively. The second stage was performed 1 month later, where the tube was inserted into the anterior chamber. There were no intraoperative or postoperative complications following staged valve implantation in the left eye. In the month between the two stages, the patient had a refraction with the pediatric ophthalmologist, which yielded a final prescription of −4.50/−0.75/180 in the right and −8.00 in the left.

One month after the second stage of the surgery, the patient returned for an EUA. The tube in the left eye was well covered, and the scleral patch graft displayed good healing. Moreover, the cupping was stable to slightly improved at 0.7 V in the left eye. IOP at this time was 30 in the right eye and 18 in the left eye. The exam also revealed an increase in the nerve size in the right eye to 0.55 V, an increase of 0.1 V. As a result of the elevated IOP and increase in cupping, the decision was made to proceed with the same procedure in the right eye.

The patient successfully underwent the first stage of the procedure, and the Ahmed tube was placed subconjunctivally in the right eye. Unfortunately, the patient underwent a period of frequent upper respiratory illnesses, which required the second stage of the procedure to be postponed due to risk with anesthesia. She finally had revision of the tube placement with insertion in the right eye 5 months after the first part of the surgery. During the revision surgery, an EUA was also performed in which IOP was measured in the eyes to be 25 in the left and 18 in the right. In these 5 months between stages of the Ahmed tube insertion in the right eye, the patient's IOP was managed with dorzolamide/timolol and latanoprost bilaterally. In her 1-week post-op check and 9 months post-EUA with CPC, the patient was doing well, and the pressure in both eyes was normal to palpation. There were no intraoperative or postoperative complications following staged valve implantation in the right eye. The patient has an extensive ocular surgical history as a result of the bilateral congenital glaucoma from SWS and ODM. She has had a trabeculotomy in both eyes at Emory, micropulse CPC on both eyes, and Ahmed tube insertion in both eyes at Rutgers New Jersey Medical School. The patient maintains a regimen of latanoprost QHS and dorzolamide/timolol BID in both eyes for further management of pressures. In the end, adequate IOP control was achieved bilaterally in this complex patient after multiple procedures including a staged insertion of Ahmed glaucoma valves (New World Medical).

## 3. Discussion

In this report, we present a rare case of SWS associated with bilateral ODM and bilateral congenital glaucoma in a 5-year-old girl in which IOP control was achieved using staged Ahmed glaucoma valve insertions. SWS can be characterized according to the Roach scale, which identifies three distinct types: Type 1—facial and leptomeningeal angiomas ± glaucoma; Type 2—facial angiomas ± glaucoma; Type 3—leptomeningeal angiomas with usually no glaucoma [15]. Based on this classification, we believe that our patient fits the Type 1 SWS classification. While the management for ODM is usually symptomatic, congenital glaucoma is a surgical disease that is often difficult to control. Medical management is not adequate to control the disease alone in most cases [16]. Many patients will need more than one procedure in their lifetime to control IOPs. Similarly, the patient presented in this report also required multiple procedures along with medical management to control her pressures. As glaucoma is often a progressive disease, it was stressed to her mother that she will need lifelong monitoring of her IOP and possible further procedures should the IOP become elevated.

Lee et al. and Gupta et al., among others, have described the use of trabeculotomy as a means to control IOP in cases of congenital glaucoma with SWS and ODM [17–20]. Here, we discuss our experience with micropulse CPC and Ahmed glaucoma valve implantation in a patient with similar findings. One of the most challenging aspects of surgery in a patient with SWS is the increased risk posed by the potential rupture of choroidal hemangiomas. Choroidal hemangiomas may be present in up to 55% of patients with ocular manifestations from SWS, according to a report by Sullivan et al., and need to be accounted for during surgical planning [6]. CPC offers a reduced risk of choroidal hemorrhage compared to incisional surgery. After discussions with the mother, she wanted to proceed with CPC first for that reason. Unsuccessful control and shared decision making with the patient's mother initiated the steps to perform Ahmed glaucoma valve surgery in both eyes. Budenz et al. and Tong et al. described cases of two-staged glaucoma implant surgeries using the Baerveldt tube in pediatric patients with SWS and congenital glaucoma [7, 21]. In fact, these were such a success that all surgery participants had achieved adequate IOP control (< 21 mmHg) within an average follow-up range of 35 months [7]. Hamush et al. reported a 79% success rate after 2 years and 30% after 5 years in their study of 11 eyes implanted with the Ahmed valve due to SWS associated with childhood glaucoma [22]. These findings supported our decision to go ahead with the valve insertion in both of our patient's eyes, and two successful procedures were performed. The multistage procedure is especially beneficial because it may be able to minimize the risk of choroidal hemorrhage in this patient population, and the valve mechanism added an extra layer of security against very low IOPs. Therefore, we recommend staged implant surgery as first-line treatment to all cases of SWS and ODM for optimal IOP control and to prevent intraoperative risks posed by choroidal hemorrhages. We believe this patient's glaucoma was caused by anterior segment dysgenesis and that ODM did not play a significant role since the trabecular meshwork was not significantly pigmented. Further studies with larger numbers of patients are necessary to elicit the long-term outcomes of glaucoma treatments in SWS patients with combined ODM.

In conclusion, we present a case of congenital glaucoma secondary to SWS and ODM in which Ahmed valve insertion successfully treated IOPs. We hope that this report can help physicians to plan for the treatment of their own patients with these disorders and to raise awareness of this co-occurrence among other ophthalmologists.

## Data Availability

Data sharing is not applicable to this article as no new data were created or analyzed in this study.
